# Towards coeliac‐safe bread

**DOI:** 10.1111/pbi.13273

**Published:** 2019-12-24

**Authors:** Zhiyong Zhang, Yiting Deng, Wei Zhang, Yongrui Wu, Joachim Messing

**Affiliations:** ^1^ Waksman Institute of Microbiology Rutgers University Piscataway NJ USA; ^2^ National Key Laboratory of Plant Molecular Genetics CAS Center for Excellence in Molecular Plant Sciences Institute of Plant Physiology & Ecology Shanghai Institutes for Biological Sciences Chinese Academy of Sciences Shanghai China; ^3^ University of the Chinese Academy of Sciences Beijing China

**Keywords:** teff, wheat, maize, α‐globulin, gluten, storage vacuole

## Abstract

Gluten‐free foods cannot substitute for products made from wheat flour. When wheat products are digested, the remaining peptides can trigger an autoimmune disease in 1% of the North American and European population, called coeliac disease. Because wheat proteins are encoded by a large gene family, it has been impossible to use conventional breeding to select wheat varieties that are coeliac‐safe. However, one can test the properties of protein variants by expressing single genes in coeliac‐safe cereals like maize. One source of protein that can be considered as coeliac‐safe and has bread‐making properties is teff (*Eragrostis tef*), a grain consumed in Ethiopia. Here, we show that teff α‐globulin3 (Etglo3) forms storage vacuoles in maize that are morphologically similar to those of wheat. Using transmission electron microscopy, immunogold labelling shows that Etglo3 is almost exclusively deposited in the storage vacuole as electron‐dense aggregates. Of maize seed storage proteins, 27‐kDa γ‐zein is co‐deposited with Etglo3. Etglo3 polymerizes via intermolecular disulphide bonds in maize, similar to wheat HMW glutenins under non‐reducing conditions. Crossing maize Etglo3 transgenic lines with *α*‐, *β*‐ and *γ‐zein* RNA interference (RNAi) lines reveals that Etglo3 accumulation is only dramatically reduced in *γ‐zein* RNAi background. This suggests that Etglo3 and 27‐kDa γ‐zein together cause storage vacuole formation and behave similar to the interactions of glutenins and gliadins in wheat. Therefore, expression of teff α‐globulins in maize presents a major step in the development of a coeliac‐safe grain with bread‐making properties.

## Introduction

Seed storage proteins (SSPs) in cereal grains not only provide major protein nutrition for humans and livestock, but also determine grain properties for processing and various uses, such as bread dough (Shewry and Halford, 2002). Based on their extraction and solubility, SSPs are commonly grouped into several classes, which include water‐soluble albumins, acid/alkaline‐soluble glutelins, saline‐soluble α‐globulins and alcohol‐soluble prolamins (Shewry and Casey, 1999). Prolamins are evolutionarily the youngest SSPs and are predominant in most cereals, except for rice and oat (Shewry and Halford, 2002; Xu and Messing, 2009). Prolamins can be divided into group I, II and III (Xu and Messing, 2009). Based on protein homology, the youngest group, group I, called α‐prolamins, originated from δ‐prolamins, which are absent in the *Pooideae* subfamily [wheat (*Triticum aestivum*), barley (*Hordeum vulgare*) and rye (*Secale cereale*)]. Although *Ehrhartoideae* [rice (*Oryza sativa*)], which is the closest subfamily to the *Pooideae*, also contains group I prolamins, but comprising only 20%–30% of the SSPs in rice grains. Among the *Panicoideae* [maize (*Zea mays*), sorghum (*Sorghum bicolor*) and little millet (*Panicum sumatrense*)], α‐prolamins represent the most abundant SSPs. Group II prolamins exist in many grass species and are composed of β‐ and γ‐prolamins in the *Panicoideae*, Ory13 and Ory16 in rice, and low‐molecular‐weight (LMW) glutenins and gliadins in the *Pooideae*. Consistent with the evolutionary relationship between subfamilies in the grasses, the Group II prolamins from the *Pooideae* and *Ehrhartoideae* cluster together, and those of *Panicoideae* fall into a separate group. The oldest prolamins are those of Group III, which only exist in the *Pooideae*. These include x‐/y‐high‐molecular weight (HMW) glutenins in wheat, D‐hordein in barley and Bra3 in *Brachypodium distachyon*. Comparative analysis of orthologous HMW genomic loci between representative grass species suggests that HMW glutenins evolved from the tandem duplication of a gene encoding an α‐globulin (Gu *et al.*, 2010; Xu and Messing, 2009).

Due to their evolutionary divergence, prolamins from different species possess unique cellular and molecular properties that have generated the extant diversity in quality and end use of cereal grains (Shewry and Halford, 2002). For instance, wheat prolamins, commonly called glutens, include HMW glutenin subunits (GSs), LMW glutenin subunits (LMW‐GSs) and gliadins. Although HMW‐GSs only comprise 5%–10% of total seed proteins, they can form polymers via intermolecular disulphide bonds as the backbone of the gluten elastomeric network associated with dough‐making properties (Shewry and Tatham, 1990; Shewry and Tatham, 1997). Therefore, HMW‐GS loci are highly related to wheat bread quality (Shewry and Halford, 2002). Because the oldest prolamins, HMW‐GSs, do not exist in other grass subfamilies, the rheological properties of other cereals are too weak to make dough‐derived foods. However, gluten also triggers an autoimmune disease, called coeliac disease, which is estimated to affect 1% of the population in North America and Europe (Gujral *et al.*, 2012). A lifelong gluten‐free diet is currently the best choice for patients with coeliac disease.

Breeding coeliac‐safe wheat is hampered by the presence of SSP genes in form of a large gene family with many variations (Zhang et al., 2013). Wheat lines with the reduction in gliadins have low levels of toxicity for patients with coeliac disease (Garcia‐Molina *et al.*, 2019; Gil‐Humanes *et al.*, 2010), although it is not known which variant is coeliac‐safe and it is not feasible to separate a mixture of so many similar proteins. The best approach to separation is expressing single genes in a related cereal that is coeliac‐safe like maize. To circumvent testing different wheat glutens, we turned to teff, an orphan grain that is used to make a type of flatbread, injera, in Ethiopia (Cochrane and Bekele, 2017). Teff belongs to the subfamily *Chlorodoideae*, which is more closely related to the *Panicoideae* than the *Pooideae* (Kellogg, 2001). The teff genome does not contain any genes that encode gluten or gluten‐like prolamins (Adebowale *et al.*, 2011; Zhang *et al.*, 2016). *In vitro* immune detection also indicates the potential safety of teff for consumption by patients who are sensitive to gluten or gluten‐like proteins in wheat, barley and rye (Spaenij‐Dekking *et al.*, 2005). To some extent, teff could be an alternative to wheat to make gluten‐free foods (Zhu, 2018).

To dissect the components in teff giving rise to injera, we examined which of the teff's SSPs could mimic wheat flour properties. Teff SSPs are mainly composed of prolamins (called eragrostis), glutelins, and albumin and α‐globulin, which account for more than 40%, 20% and 11% of the total proteins, respectively (Adebowale *et al.*, 2011). When eragrostins are extracted with 70% ethanol and separated by SDS‐PAGE, the migration pattern is almost identical to that of species of the subfamily *Panicoideae*, such as maize and sorghum (Zhang *et al.*, 2016). Phylogenetic analysis further indicates that different eragrostins cluster with the corresponding zeins into groups I and II and are clearly separated from wheat glutenins and gliadins (Figure S1). However, glutelins cannot account for teff grain bread‐making properties because rice glutelins do not confer bread‐making qualities (Takaiwa *et al.*, 1999). A distinct difference between maize and teff is the level of α‐globulins in the seed. Because the content of α‐globulin in most cereals is too low to be detected by SDS‐PAGE of total grain proteins, they limit the processing and utilization of most grains. Rice and teff are exceptional in their relatively high abundance of α‐globulins, which are comparable to that of HMW glutenin in wheat seeds. However, rice flour cannot form dough to make bread like wheat and teff. First, rice major SSPs consist of glutelins, which account for about 60% of total seed proteins, whereas prolamins comprise 20%–30% of total seed proteins (Takaiwa *et al.*, 1999). Second, rice α‐globulin is a monomeric protein in the endosperm because all eight cysteine residues form intramolecular disulphide bonds (Kawagoe *et al.*, 2005). In contrast to HMW glutenin, rice α‐globulin cannot provide the backbone to interact with other SSPs. Third, gluten aggregates into sizeable storage vacuoles in wheat endosperm cells, which are associated with the property of viscoelasticity (Shewry *et al.*, 1995). However, no sizeable storage vacuoles are present in rice endosperm cells (Bechtel and Juliano, 1980; Krishnan *et al.*, 1986). Therefore, rice α‐globulin and HMW glutenin are different at the molecular and cellular levels.

Therefore, we used a transgenic approach to overcome the divergence of SSPs during speciation. Because of the different bread‐making qualities of teff and maize despite their similar prolamin content, we examined whether the introduction of teff α‐globulins into maize could mimic the cellular properties of wheat endosperm, which indeed was the case. Therefore, this reverse evolutionary approach represents the first step towards the development of a coeliac‐safe alternative to wheat flour.

## Results

### Differences among teff α‐globulins from maize, rice and wheat

α‐globulin is a typical ABC domain‐containing SSP (Shewry and Tatham, 1990). The ABC regions of α‐globulins that contain the eight cysteine residues are highly conserved among different cereals, although their flanking sequences have no significant similarity (Figure S2). Due to their low content in most grains except for rice and teff, limited knowledge exists concerning the cellular effects of α‐globulins (Woo *et al.*, 2001). To date, the only characterized rice α‐globulin is a monomeric protein in which all eight cysteine residues form intramolecular disulphide bonds. Similar to rice, maize and wheat α‐globulins also contain an even number of cysteine residues, suggesting that they are monomeric proteins. Teff is the only cereal that contains four α‐globulins; namely Etglo1, Etglo2, Etglo3 and Etglo4 (Zhang *et al.*, 2016). Similar to maize, teff arose by allotetraploidization, where Etglo1 and Etglo4 derived from one progenitor and Etglo2 and Etglo3 from the other. Etglo1 and Etglo4 share 94% amino acid sequence identity and their respective genes share 95% nucleotide sequence identity, and Etglo2 and Etglo3 share 81% amino acid sequence identity and 84% nucleotide sequence identity. In addition to the eight shared cysteine residues, teff α‐globulins have four to seven additional cysteine residues, which could form intermolecular disulphide bonds (Table S1). In this study, we chose the largest α‐globulin, Etglo3, with an uneven number of cysteine residues to investigate the molecular and cytological characteristics of teff α‐globulins in maize endosperm cells.

### Expression of the teff α‐globulin gene *Etglo3* in maize endosperm

**Figure 1:**
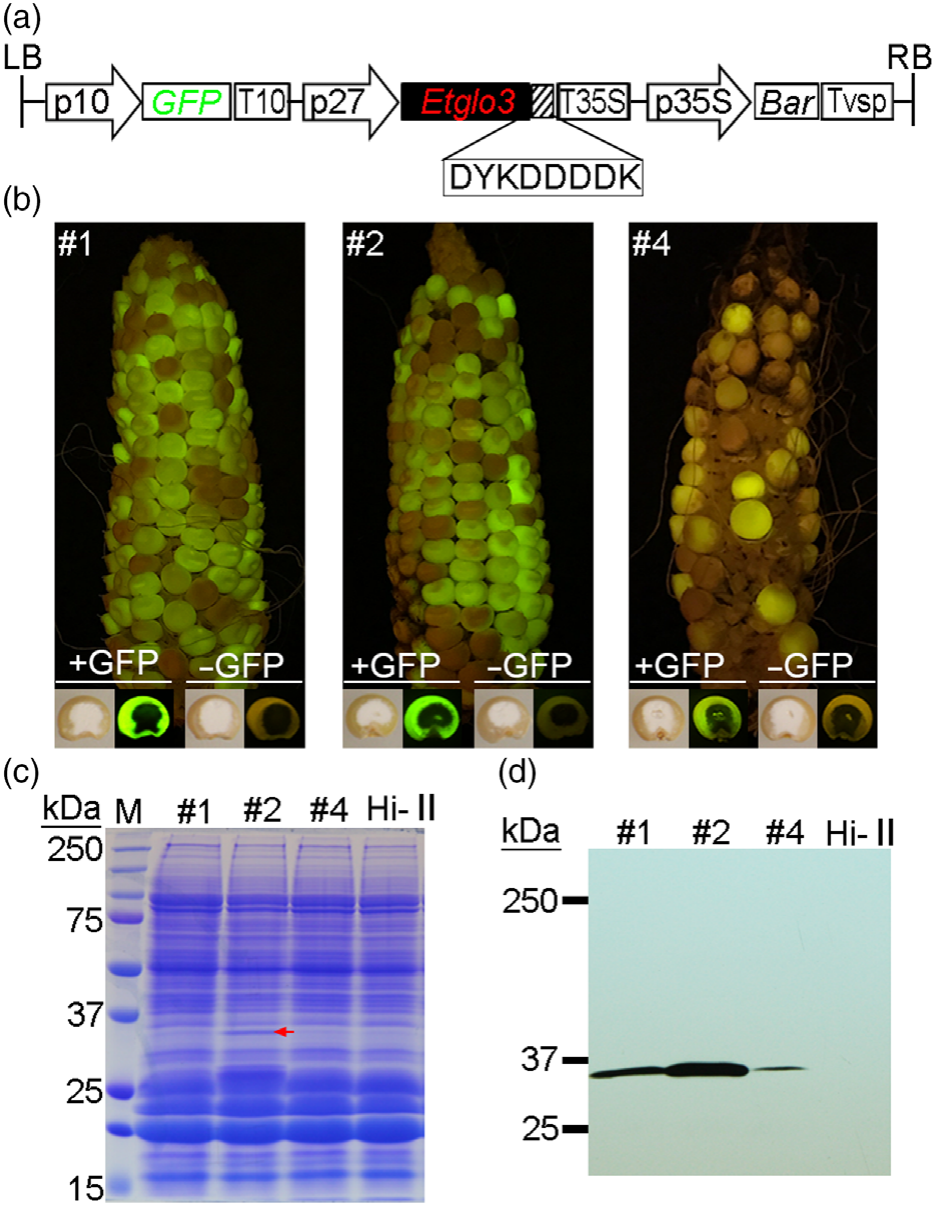
Generation of Etglo3 transgenic maize plants by *Agrobacterium*‐mediated transformation. (a) Schematic diagram of the Etglo3 transgene construct. The left and right borders (LB and RB) of the T‐DNA binary vector flank the region that was transformed into maize; p10 and p27 represent 10‐kDa δ‐zein and 27‐kDa γ‐zein promoters, respectively; GFP, green fluorescent protein; T10, 10‐kDa δ‐zein terminator; T35S, 35S terminator; p35S, 35S promoter; Bar, bialaphos‐resistance gene; Tvsp, soybean vegetative storage protein terminator. (b) Cobs and transverse kernels of primary transformants (T1) of three independent transgenic lines (#1, #2 and #4) under fluorescent/white light. (c, d) SDS‐PAGE and immunoblotting of Etglo3 protein in total proteins from mature seeds of Etglo3 transgenic plants. Twenty micrograms of total protein per line was loaded onto two SDS‐PAGE gels. The left gel was stained with Coomassie Brilliant Blue, and the right gel was immunoblotted with the anti‐FLAG antibody. The red arrow in panel (c) indicates the visible accumulation of Etglo3 protein in Etglo3#2.

To study the molecular and cytological function of Etglo3, we created maize transgenic lines via *Agrobacterium tumefaciens*‐mediated transformation. The binary vector contained an *Etglo3* expression cassette under control of the maize endosperm‐specific *27‐kDa γ‐zein* promoter, the seed‐visible marker GFP (green fluorescent protein) driven by endosperm‐specific *10‐kDa δ‐zein* promoter and a FLAG tag at the C terminus of Etglo3 for protein detection and cellular immune localization (Figure 1a). This resulted in three independent transgenic events (named Etglo3#1, Etglo3#2 and Etglo3#4) (Figure 1b). PCR analysis of genomic DNA showed that the *Etglo3*, *GFP* and *Bar* genes were present in the three transgenic lines of T_1_ seeds with a GFP signal but not in seeds without the GFP signal; therefore, GFP is a stable selection marker for the presence of the *Etglo3* transgene in seeds (Figure S3A).

**Figure 2:**
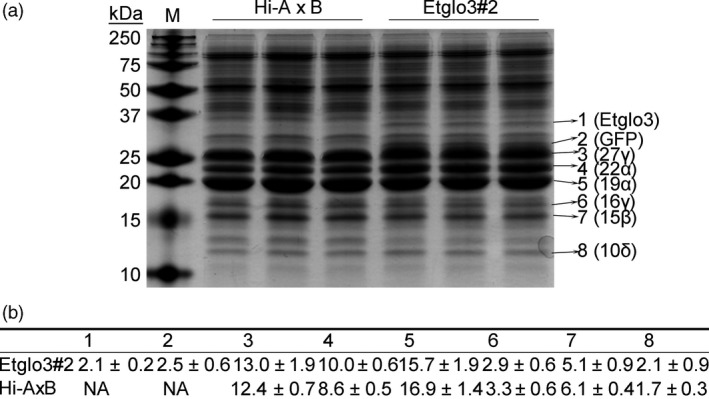
Quantification of Etglo3 and zein accumulation. (a) SDS‐PAGE of total proteins from mature seeds of Etglo3#2 and wild‐type Hi‐A×B. The SDS‐PAGE gel was 15% (w/v). The eight numbers on the right indicate the following bands on the gel: 1, Etglo3; 2, GFP; 3, 27‐kDa γ‐zein (27γ); 4, 22‐kDa α‐zein (22α); 5, 19‐kDa α‐zein (19α); 6, 16‐kDa γ‐zein (16γ); 7, 15‐kDa γ‐zein (15β); and 8, 10‐kDa δ‐zein (10δ). (b) Quantification of the eight bands in panel (a) by AlphaView software. The percentage per protein refers to protein accumulation compared to the total protein. The amounts of proteins ± SD from three replicates of Etglo3 transgenic plants and Hi‐A×B in the above gel are shown.

Transgenic and non‐transgenic seeds from T_1_ transgenic cobs had a similar kernel texture (Figure 1b). A visible band of about 30 kDa was detected in SDS‐PAGE of total proteins of mature T_1_ Etglo3#2 transgenic seeds, which corresponds to the molecular weight of the Etglo3 protein (Figure 1c). The accumulation of Etglo3 in transgenic seeds was confirmed by immunoblotting using the primary antibody against the FLAG tag (Figure 1d). Consistent with the transcript level determined by real‐time quantitative PCR (Figure S3B), Etglo3#2 contained the highest protein abundance of all three transgenic lines and Etglo3 accumulated to ~2.1% of total seed protein, similar to the content of 10‐kDa δ‐zein but lower than that of 27‐kDa γ‐zein, which accumulated to 13% of total seed protein (Figure 2). Although this level of expression of Etglo3 is much lower than it is in teff and not sufficient yet to achieve bread‐making properties of maize flour, it should provide a critical test case to study its impact on maize endosperm. We used Etglo3#2 for all subsequent experiments.

### *Etglo3* transgene contains storage vacuoles with electron‐dense aggregates in starchy endosperm cells

**Figure 3:**
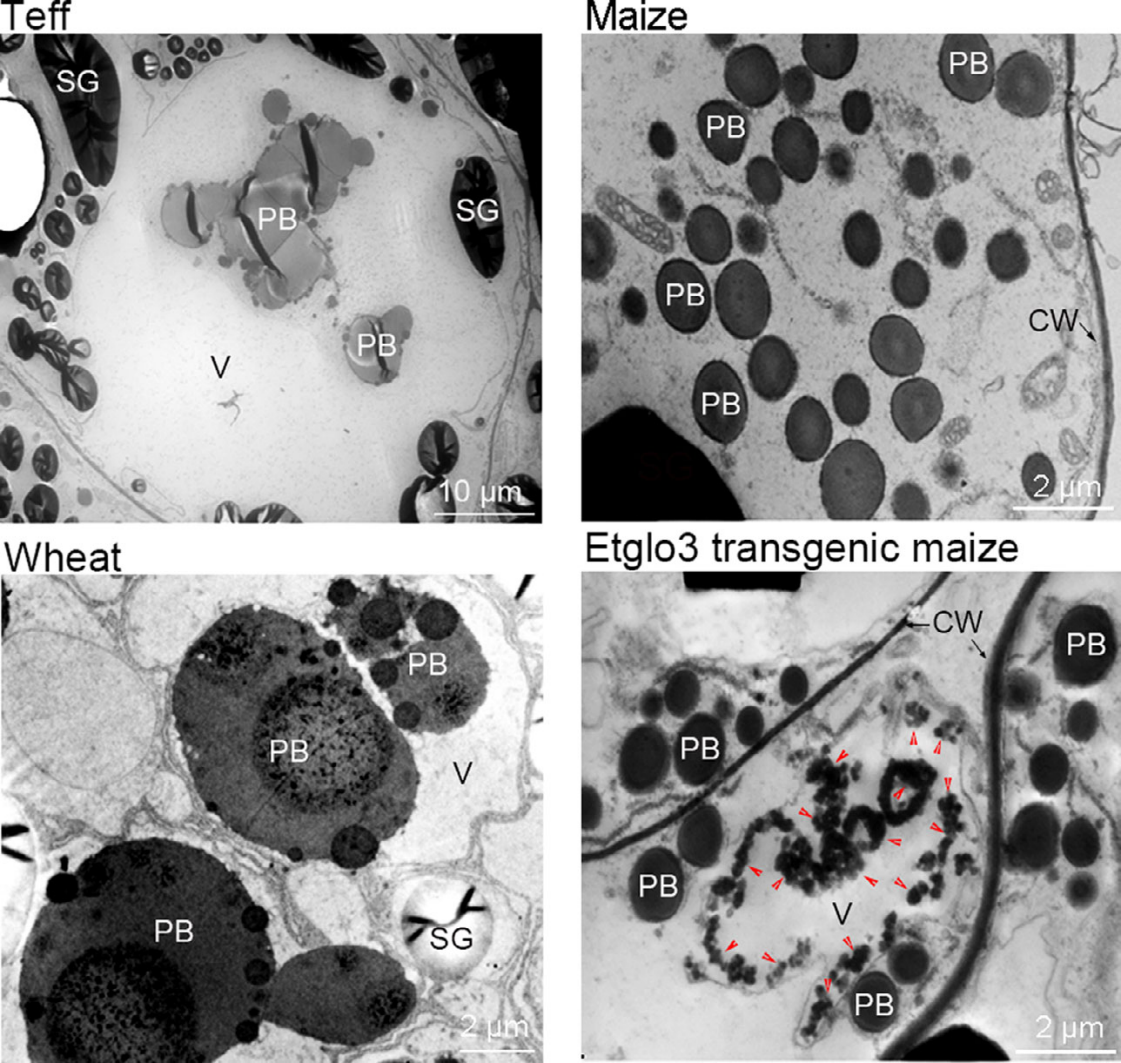
Transmission electron micrographs of developing endosperm cells of teff, wheat, maize and Etglo3 transgenic maize. SG, starch granule; PB, protein body; V, vacuole; CW, cell wall. In the panel of Etglo3 transgene maize, red arrowheads indicate electron‐dense aggregates.

The properties of major storage proteins mostly affect the storage protein compartments within developing endosperm cells of different grains. Although teff prolamins and zeins are similar in size and sequence, the storage protein compartment morphology in teff endosperm cells differed from that in maize. Teff storage proteins accumulated in protein bodies (PBs), which aggregated into storage compartments within the endosperm cell vacuole, whereas all starch granules were excluded from the vacuole (Figure 3). Consistent with previous observations (Shewry *et al.*, 1995), wheat endosperm also contains fused PBs within the vacuole, but these are morphologically different from those of teff (Figure 3). By contrast to PBs in teff and wheat, maize PBs are small (1–2 μm in diameter), spherical, uniform and abundant. Although maize endosperm cells have no PB‐containing vacuoles, a few protein storage vacuoles (PSVs) are present (Arcalis *et al.*, 2010; Reyes *et al.*, 2011). These PSVs are smaller in maize than in teff and wheat but similar in size to maize PBs. Furthermore, electron‐dense α‐globulin inclusions were observed in PSVs but not in PBs.

Unlike non‐transgenic, Etglo3 transgenic lines produced many electron‐dense aggregates within a large vacuole in starchy endosperm cells, whereas the large vacuole contained no PBs (Figure 3). The morphology and size of PBs were similar in non‐transgenic and Etglo3 transgenic lines. These electron‐dense aggregates were not surrounded by a discernable membrane and resembled α‐globulin inclusion in maize PSVs. The electron‐dense aggregates were dispersed in the periphery of non‐transgenic maize PSVs but also Etglo3 transgenic vacuoles. Although maize Etglo3 transgenic cells produced a large storage vacuole, it had a different morphology and composition to that of teff and wheat. This suggested that vacuole formation was due to the accumulation of the teff α‐globulin Etglo3.

### Localization of Etglo3 and 27‐kDa γ‐zein in electron‐dense aggregates and PBs

**Figure 4:**
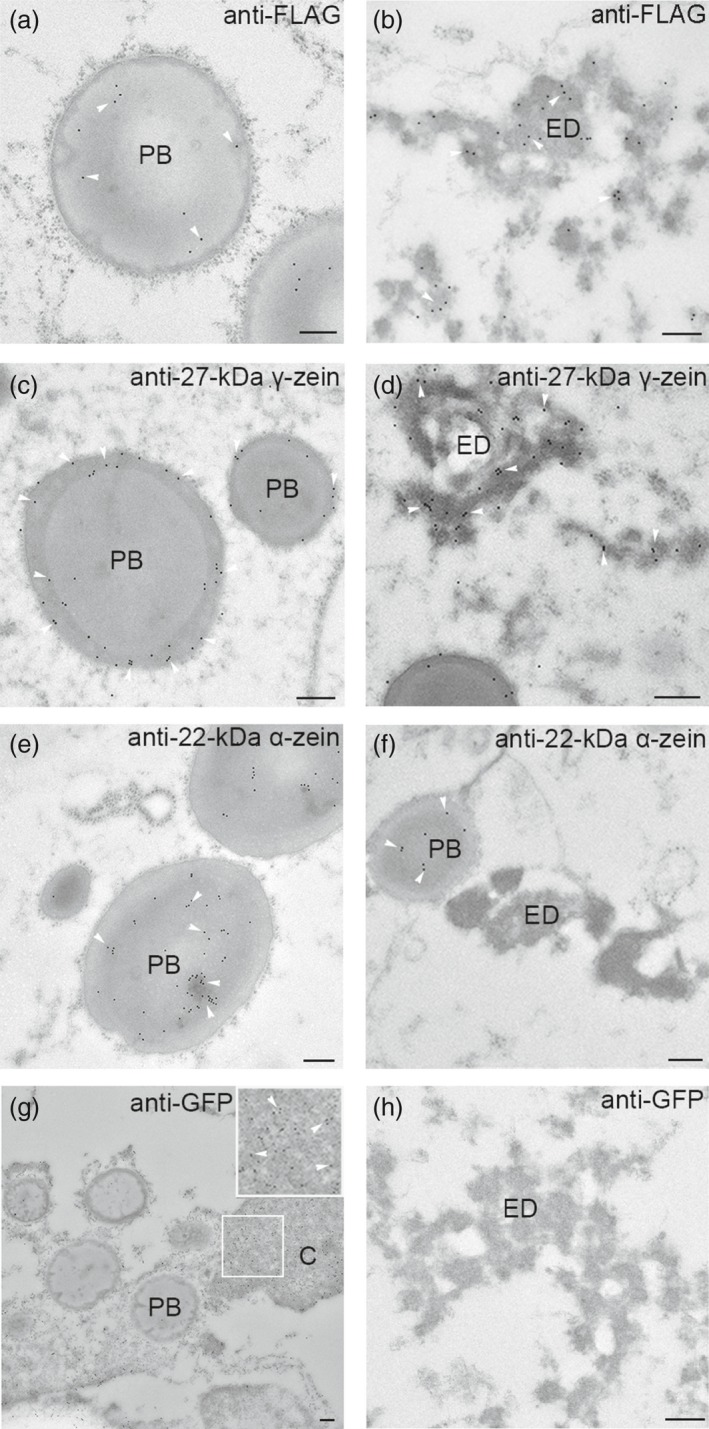
Subcellular localization of Etglo3, 27‐kDa γ‐zein, 22‐kDa α‐zein and GFP by immunogold labelling TEM in the 20‐DAP starchy endosperm cells of Etglo3 transgenic plants. (a) and (b) show subcellular localization of Etglo3 by labeling the FLAG tag. (c) and (d) show subcellular localization of 27‐kDa γ‐zein. (e) and (f) show subcellular localization of 27‐kDa α‐. (g) and (h) show subcellular localization of GFP as the control. White arrowheads indicate immunogold particles. PB, protein body; ED, electron‐dense aggregates. In (g), the cytoplasmic (C) area marked with a white frame is enlarged and immunogold signals are highlighted with arrowheads. Bars = 200 nm in all images.

To analyse the subcellular localization of storage proteins, we conducted transmission electron microscopy (TEM) using immunogold labelling of primary antibodies against the FLAG tag and α‐/γ‐zein in the developing endosperm of Etglo3 transgenic line. Because the FLAG tag was fused to the C terminus of Etglo3 (Figure 1a), immunogold labelling against the FLAG tag detected the subcellular localization of Etglo3. Etglo3 localized not only to PBs but also to the electron‐dense inclusions, whereas it was distributed around the periphery of PBs (Figure 4a,b). Furthermore, by quantifying the number of gold particles within electron‐dense aggregates and PBs, nearly 90% of Etglo3 localized to the electron‐dense inclusions and 10% to PBs (Figure S4). This is similar to previous observations for maize α‐globulin, which mainly localizes to the electron‐dense inclusions of PSVs and partly to the periphery of PBs (Arcalis *et al.*, 2010).

Immunogold labelling with primary antibodies against zein proteins demonstrated that 27‐kDa γ‐zein was present at the periphery of PBs and within electron‐dense aggregates, although 22‐kDa α‐zein was only located within the centre of PBs (Figure 4c–f). The subcellular co‐localization of Etglo3 and 27‐kDa γ‐zein suggests that the two storage proteins might interact with each other. Besides a role in the initiation of PB formation, 27‐kDa γ‐zein probably also contributes to the formation of storage vacuoles in the starchy endosperm of *Etglo3* transgenic plants. Immunogold labelling of GFP as the control revealed that GFP was exclusively present in the cytoplasm, but not in PBs and electron‐dense inclusions (Figure 4g,h).

### Polymerization of Etglo3 proteins

**Figure 5:**
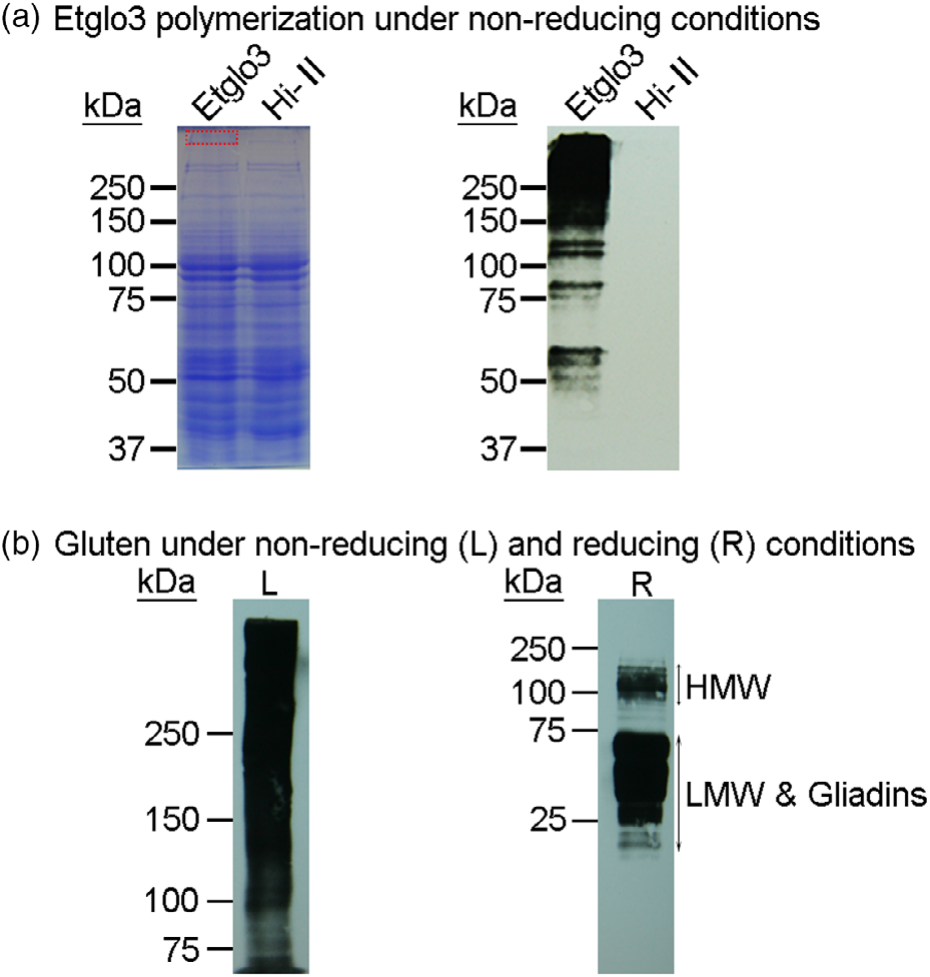
Immunoblotting detection of Etglo3 and gluten polymerization. (a) immunoblotting of total proteins of mature seeds of Etglo3 transgenic plants (Etglo3) and Hi‐A×B (Hi‐II) with an anti‐FLAG antibody under non‐reducing conditions. Total seed proteins were extracted and separated on the same two SDS‐PAGE gels (8% w/v). The left gel was stained with Coomassie Brilliant Blue (CBB), and the right gel was immunoblotted. Non‐reducing conditions are those in which the extraction and loading buffer did not contain β‐mercaptoethanol (reducing reagent). A piece of gel marked by the red rectangle on the top of CBB‐stained SDS‐PAGE was excised to perform LC‐MS. The top 30 identified proteins are listed in Table S2. (b) Immunoblotting of total wheat seed proteins with an anti‐gluten antibody. This antibody recognizes all wheat glutens. On the left (L), 5% (w/v) SDS‐PAGE was used to separate the total proteins of wheat mature seeds under non‐reducing conditions (without β‐mercaptoethanol in the extraction and loading buffer). On the right (R), 15% (w/v) SDS‐PAGE was used to separate the total proteins from wheat mature seeds under reducing conditions (containing 5% β‐mercaptoethanol in the loading buffer). The amount of protein loaded in each lane was 20 µg.

Because the Etglo3 protein contains an uneven number of cysteine residues, we assumed that it might form polymers via intermolecular disulphide bonds, similar to HMW glutenins. We first employed liquid chromatography–mass spectrometry (LC‐MS) to analyse a narrow slice of SDS‐PAGE gel, representing the putative polymeric Etglo3 protein derived from total seed proteins under non‐reducing conditions (Table S2). In addition, immunoblotting using the primary anti‐FLAG antibody was used to detect the polymerization of Etglo3 protein in transgenic seeds under non‐reducing conditions (Figure 5a), with an antibody against whole gluten proteins under reducing and non‐reducing conditions as a control. This showed that the Etglo3 protein polymerizes with a pattern that resembles the continuous network of gluten polymerization (Figure 5b). To further test the importance of an uneven number of cysteine residues on polymerization, we also analysed transgenic maize plants containing teff α‐globulin Etglo4 with an even number of cysteine residues. Unlike Etglo3, Etglo4 did not polymerize under non‐reducing conditions (Figure S5). Therefore, the number of cysteine residues in an α‐globulin protein was needed for its polymerization.

### Interaction of Etglo3 with zein proteins

**Figure 6:**
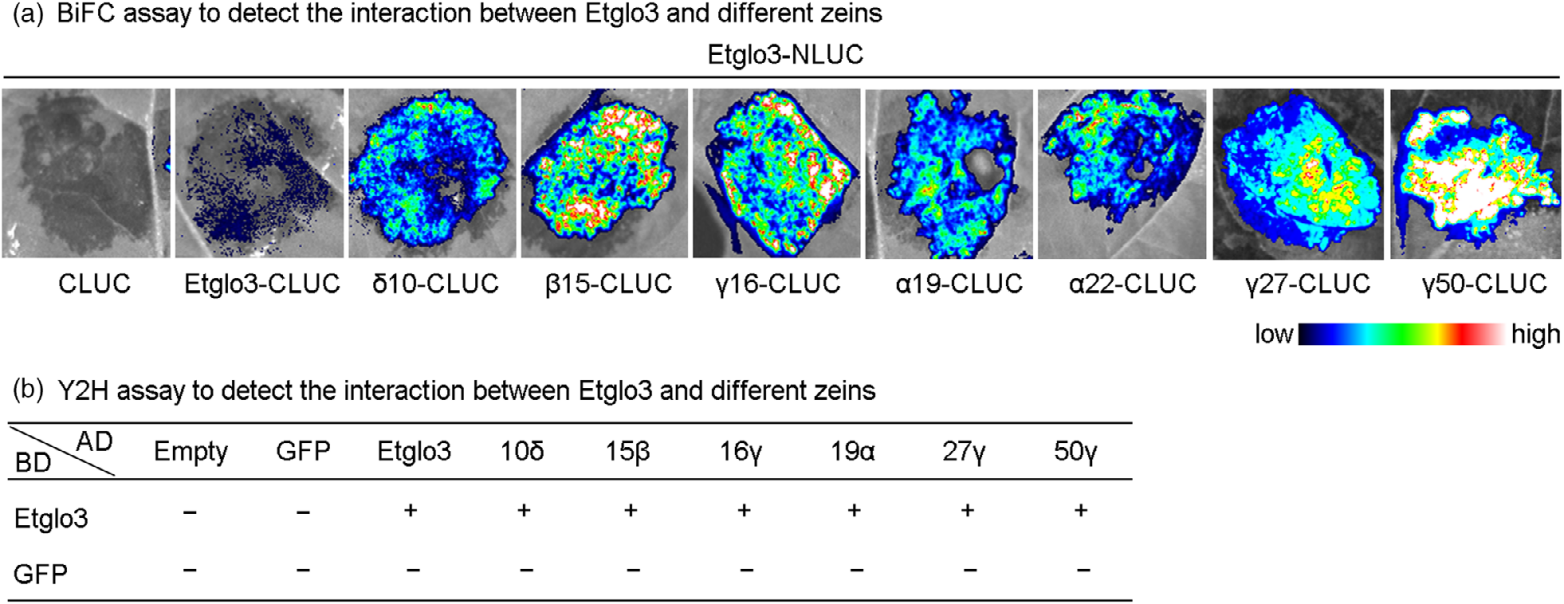
Bimolecular fluorescence complementation (BiFC) (a) and yeast two‐hybrid (Y2H) assay (b) For Review Only detection of the interaction between Etglo3 and different zeins. The fluorescence signal intensities represent the interaction in (a). The detailed Y2H results are depicted in Figure S6. ‘+’ represents interaction, and ‘−’ represents no interaction.

Based on the polymerization of α‐globulin with an uneven number of cysteine residues and its direct evolutionary relationship with HMW glutenin, we hypothesized that teff α‐globulins are equivalent to HMW glutenin as the molecular backbone for the interactions between prolamins. Because zeins are alcohol‐soluble and cannot be extracted with buffer for co‐immunoprecipitation, we employed instead bimolecular fluorescence complementation and yeast two‐hybrid assays to address the potential interaction between Etglo3 and different zein proteins in maize endosperm. These approaches showed that Etglo3 could interact with itself and different zeins (Figure 6 and Figure S6), suggesting that the polymerized structure of α‐globulin has a similar function to HMW glutenin in providing the molecular backbone for prolamin interaction.

**Figure 7:**
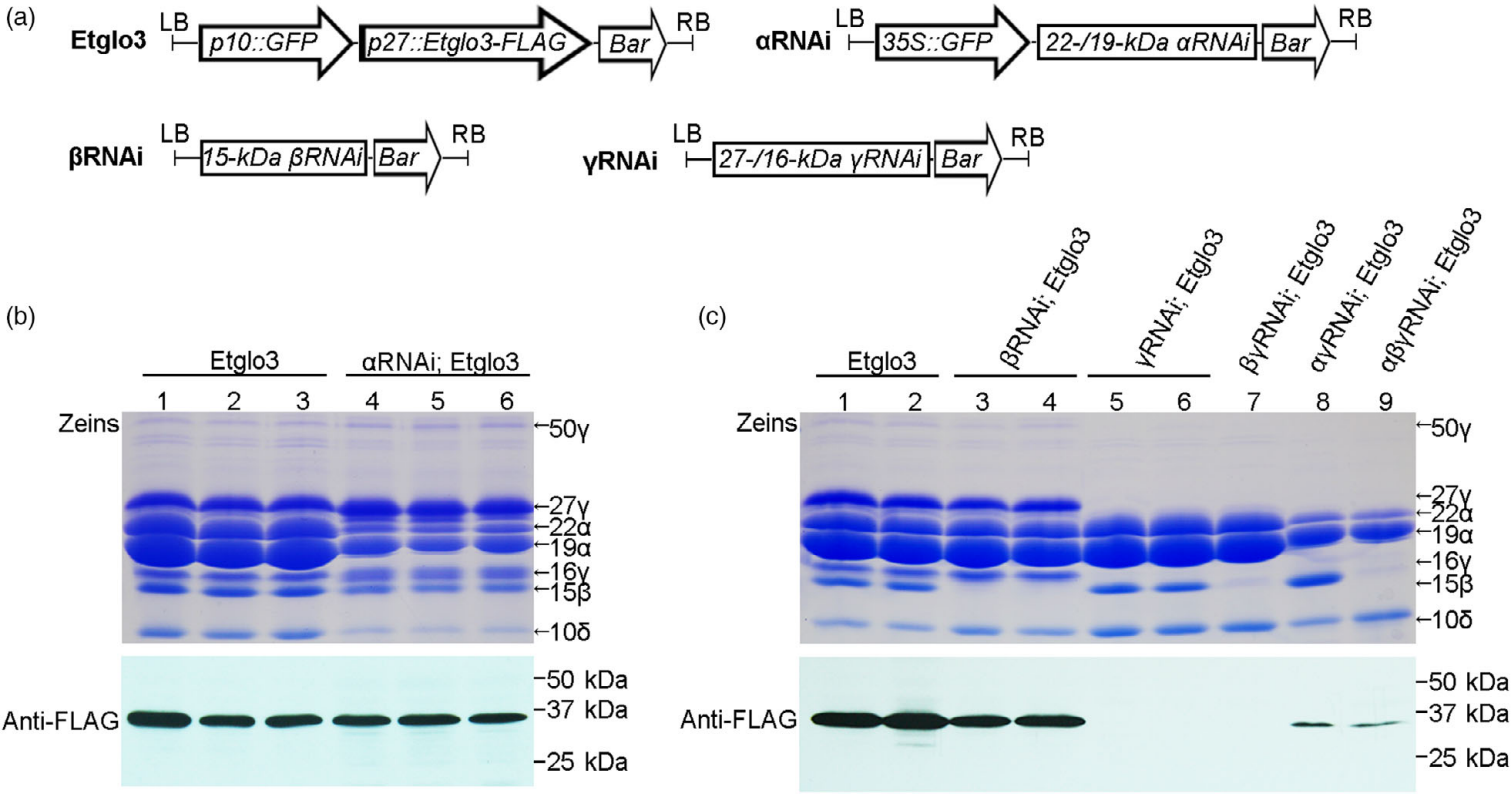
The genetic crosses between Etglo3 transgene and α‐/β‐/γ‐zein RNAi and combined RNAi lines. (a) Schematic diagram of the transformation constructs used in this study. (b,c) Immunoblotting of Etglo3 in mature seeds of progeny of the cross between Etglo3 transgene and the α‐zein RNAi line (b), and between β‐/γ‐zein and their combined RNAi line (c). In (b) and (c), Coomassie Brilliant Blue‐stained gels of zein proteins were used for the genotypes indicated, and immunoblotting with the anti‐FLAG antibody was performed using the corresponding non‐zein proteins.

To test the effects of different zein proteins on Etglo3 accumulation, we conducted genetic crosses between *Etglo3* transgenic maize lines and RNAi lines for *α‐*, *β‐*, *γ‐zein* and their combinations. Total seed protein was extracted from the mature dry seeds of progeny from these crosses. Immunoblotting with an anti‐FLAG antibody detected the accumulation of Etglo3 protein in the genetic background of *α*‐, *β*‐ and *γ‐zein* RNAi (Figure 7). Although there was no visible change in Etglo3 protein accumulation in the *α*‐ or *β‐zein* RNAi background, Etglo3 protein almost disappeared in the *γ‐zein* RNAi background (Figure 7b,c). The 27‐kDa γ‐zein was essential for the initiation of PB formation (Coleman *et al.*, 1996; Lending and Larkins, 1989; Mainieri *et al.*, 2014). This suggested that 27‐kDa γ‐zein probably together with 16‐kDa γ‐zein was critical for the accumulation of Etglo3 in maize endosperm cells.

### Autophagy probably involves the formation of storage vacuoles containing electron‐dense aggregates in Etglo3 transgenic endosperm

Although teff and wheat produce large PB‐containing vacuoles in endosperm cells, the molecular mechanism for formation of this type of vacuole is unknown. Because *Etglo3* transgenic endosperm produced a storage vacuole that contained teff α‐globulin and γ‐zein, it can be used to study storage vacuole formation. In maize endosperm, PBs are predominantly present in starchy endosperm cells and PSVs exist in aleurone cells and a few peripheral starchy endosperm cells (Woo *et al.*, 2001), although the morphology and size of PSVs were different from those of the storage vacuoles in teff, wheat and Etglo3 transgenic plants. Autophagy is involved in the formation of PSVs in maize aleurone cells (Reyes *et al.*, 2011). The *Etglo3* transgenic plants provide an opportunity to analyse which genes are potentially involved in the formation of storage vacuoles that contain electron‐dense aggregates in starchy endosperm cells. It has been proposed that the molecular chaperone BiP (binding protein) facilitates prolamin folding and assembly into PBs in the endosperm cells of maize and rice (Li *et al.*, 1993; Zhang and Boston, 1992). Because BiP2 (GRMZM2G114793) and BiP3 (GRMZM2G415007) homologs were enriched in maize PB proteomes (Wang *et al.*, 2016), we analysed their expression in *Etglo3* transgenic and non‐transgenic control plants. The expression of *BiP2* was up‐regulated more than twofold in the developing endosperms of Etglo3 transgenic plants (Figure S7).

Another molecular chaperone, protein disulphide isomerase (PDI), is important for disulphide bond formation in the endoplasmic reticulum (ER) of maize, rice and wheat (Li and Larkins, 1996; Onda and Kobori, 2014; Shimoni *et al.*, 1995). We analysed the expression of maize *PDI‐like* (*PDIL*) family genes *in silico* using the database maizeGDB. Three out of the 12 identified *PDIL* genes (*PDIL1;1*, *PDIL2;3* and *PDIL6*) were up‐regulated 1.5‐fold to twofold in the *Etglo3* transgenic line (Figure S7). In rice, PDIL1;1 and PDIL2;3 favour the folding arrangement of vacuole‐targeted storage proteins (glutelin and α‐globulin) and prolamins, respectively (Kim *et al.*, 2012; Onda *et al.*, 2011). The results suggested that Etglo3 protein migrated into the PB ER and interacted with these molecular chaperones.

Because atypical autophagy should involve the delivery of prolamins to PSVs in maize aleurone cells (Reyes *et al.*, 2011), transcripts of *AUTOPHAGY‐RELATED* (*ATG*) family genes were detected in the developing endosperm of *Etglo3* transgenic plants, in which the expression of *ATG8* was not altered. This observation was consistent with a previous finding, where ATG8‐dependent macro‐autophagy was not related to PSV formation in maize endosperm cells (Reyes *et al.*, 2011). However, *ATG10* expression was up‐regulated more than threefold in *Etglo3* transgenic plants (Figure S7). This suggested that autophagy probably involved the trafficking of Etglo3 from the ER to the storage vacuole. In maize aleurone cells, PSV formation was autophagy‐dependent, whereas the Golgi‐dependent pathway was dominant in delivering storage proteins to the PSV in rice endosperm cells (Fukuda *et al.*, 2016; Tian *et al.*, 2013). Previous studies had demonstrated that Golgi‐dependent PSV formation probably also occurred in maize and wheat endosperm cells (Reyes *et al.*, 2011; Tosi, 2012). The coat protein complex II (COPII) was required for the transportation of glutelin and α‐globulin from the ER to the Golgi during rice PSV formation. Rice small GTPase (Sar1) and Golgi transport 1 (GOT1) mediated the Golgi‐dependent pathway (Fukuda *et al.*, 2016; Tian *et al.*, 2013). We also detected expression of maize *Sar1* and *GOT1* homologs in *Etglo3* transgenic plants by real‐time qPCR, and their expression level in endosperm cells was the same as in the non‐transgenic control (Figure S7). This suggests that autophagy but not the Golgi‐dependent pathway is involved in the formation of the large storage vacuole in endosperm cells of *Etglo3* transgenic plants.

## Discussion

To our knowledge, this is the first account of the convergent relationship between α‐globulin and HMW glutenin at the molecular and cellular levels. Previous phylogenetic evidence suggested that HMW glutenins, the oldest prolamins, evolved from the duplication of an α‐globulin gene (Xu and Messing, 2009). The HMW‐GSs in gluten can form polymers via intramolecular disulphide bonds to produce a backbone for interactions with LMW glutenins and gliadins. This plays an important role in PSV formation by merging and collapsing PB aggregates in wheat endosperm (Rubin *et al.*, 1992). These molecular and cellular properties of HMW glutenins confer bread‐making quality to wheat flour. However, to date, it is not known whether α‐globulins can function similarly in other species. Because the content of α‐globulin in most cereal grains such as maize is too low to be visible in Coomassie Brilliant Blue‐stained protein gels (Woo *et al.*, 2001), it likely has negligible effects.

The divergence in the primary structure of α‐globulin proteins also indicates that their molecular and cellular properties differ among species. This can be exemplified by two well‐studied α‐globulins. First, α‐globulin from rice, which has poor bread‐making quality, is encoded by a single‐copy gene but accounts for 4%–15% of the total SSPs (Krishnan and White, 1995). A loss‐of‐function α‐globulin mutant is mainly affected in PSV formation in rice endosperm (Lee *et al.*, 2015). Rice α‐globulin contains the eight conserved cysteine residues that are shared by all α‐globulins (Figure S2). These cysteine residues form four pairs of intramolecular disulphide bonds and ensure that rice α‐globulin remains a monomeric SSP (Kawagoe *et al.*, 2005). In rice grains, glutelins but not prolamins are the most abundant SSPs. In addition to prolamins, rice α‐globulins are synthesized within the PB‐ER lumen and are transported into the PSV together with glutelins (Washida *et al.*, 2009; Washida *et al.*, 2012); therefore, there is no molecular basis for the interaction between α‐globulin and prolamins in rice.

The second example concerns eragrostins from teff, which do not contain gluten‐like proteins but can be used to make flatbread. The four duplicated teff α‐globulins and albumins account for ~11% of total seed proteins (Adebowale *et al.*, 2011). Based on phylogenetic analysis of SSP, we hypothesize that teff α‐globulins might function similarly to HMW glutenin in wheat. It appears that a critical distinguishing feature between rice and teff might be the number of α‐globulin cysteine residues. We investigated this via a gain‐of‐function experiment by expressing a teff α‐globulin in maize endosperm. We observed the same aggregation, where an uneven number of cysteine residues in α‐globulins mimic the wheat glutenins. In wheat, HMW glutenin interacts with LMW glutenin and gliadins, whereas Etglo3 interacts with γ‐zeins in maize, resulting also in the formation of storage vacuoles in maize starchy endosperm cells. This type of storage vacuole is common to wheat endosperm cells; massive aggregated PBs also exist in teff endosperm cells (Figure 3) (Shewry *et al.*, 1995; Zhang *et al.*, 2016). Interestingly, of all prolamins in maize the γ‐zeins are the closest to the gliadins in wheat (Xu and Messing, 2009), where we have also observed direct interactions. However, the γ‐zeins have diverged from the gliadins to a degree that they cannot substitute for them to achieve bread‐making properties, although, unlike α‐zeins, their N‐terminal part comprises seven Cys residues, providing the molecular basis of the interaction with Etglo3. The remaining ABC domain functions in vacuole secretion (Mainieri *et al.*, 2014; Pedrazzini *et al.*, 2016). Because 27‐kDa γ‐zein is the most abundant among all three γ‐zeins (Figure 2), it has a critical function in the initiation of PB formation. Therefore, despite promotion of storage vacuole formation in maize endosperm by expressing α‐globulin, PBs still remain outside of storage vacuoles in Etglo3 transgenic endosperm (Figure 3). One solution could be the substitution of the 27‐kDa γ‐zein with gliadin expression in combination with Etglo3. An additional advantage could be that a proper selection of a single gliadin that could not only provide bread‐making properties to maize, but also a coeliac‐safe combination. Meanwhile, the content of each SSP is closely related with its effect on total SSPs and grain properties. For instance, 5%–10% of HMW glutenins in wheat total SSPs is essential for dough‐making properties. The remaining challenge would be the increased expression of Etglo3 and the selected gliadin in maize with simultaneous reduction in zein levels. The latter rebalancing of the storage of reduced nitrogen had already been demonstrated (Wu and Messing, 2012). Given these common structural features, reverse evolution could conceivably play an important role in the engineering of novel cereal grains with improved nutritional quality.

## Experimental procedures

### Plasmid construction and maize transformation

The teff α‐globulin genes of *Etglo3* and *Etglo4* were amplified from teff genomic DNA. The 3ʹ terminus for each gene was linked to a short nucleotide sequence encoding the short FLAG tag peptide (DYKDDDDK). The *Etglo3* and *Etglo4* genes were expressed under control of the *27‐kDa γ‐zein* promoter and the *Cauliflower Mosaic Virus* (CaMV) *35S* terminator. The expression cassettes were then transformed into the binary vector pTF102. The transgenic vector also contained the gene conferring resistance to the herbicide bialaphos (*Bar*) and *GFP* as the selection markers for the transgene. The transgenic vector was transformed into *Agrobacterium tumefaciens* strain EHA101 as previously described (Frame *et al.*, 2002). The F_1_ hybrid of inbred maize lines Hi‐A and ‐B was used as the genetic material for *Agrobacterium*‐mediated transformation. All primers used for plasmid construction and transgenic identification are listed in Table S3.

### Crosses between *Etglo3* transgenic line and *zein* RNAi lines

The *α‐*, *β‐* and *γ‐zein* RNAi lines from previous studies (Wu and Messing, 2010; Wu and Messing, 2011) were crossed to the *Etglo3* transgenic line. The *α‐zein* RNAi line is in the Hi‐A×B genetic background, and the *β‐/γ‐zein* RNAi line possesses the genetic background of inbred line A654. Considering the different genetic backgrounds, heterozygous *α‐*, *β‐* and *γ‐zein* RNAi lines were used for crosses. The expression of *Etglo3* and *GFP* in the kernels of the same segregating ears was then analysed in the different *zein* RNAi backgrounds. The *α‐zein* RNAi and *Etglo3* transgenic lines share the same genetic background as Hi‐A×B; thus, these crosses could be individually analysed. The genotype and phenotype of the kernels in the different *zein* RNAi backgrounds were analysed via protein SDS‐PAGE and a white/fluorescent lightbox. All the genetic materials in this study were grown in the greenhouse and in the field of the Waksman Institute in New Jersey.

### Extraction of total RNA, reverse transcription and real‐time quantitative PCR

Total RNA of endosperms at 20 days after pollination (DAP) was extracted using TRIzol reagent (Invitrogen, Carlsbad, CA, USA) and purified with the RNeasy Mini Kit combined with DNase 1 digestion (Qiagen, Germantown, MD, USA). The SuperScript III First‐Strand Kit (Invitrogen) was used for reverse transcription according to the manufacturer's instructions. PowerUp SYBR Green Master Mix (Thermo Fisher Scientific, Waltham, MA, USA) was used to perform the real‐time qPCR in the Illumina Eco real‐time qPCR system. The expression level of *Etglo3* was analysed by the comparative ∆∆CT method, and the maize *ACTIN* gene was used as the reference gene. All primers are listed in Table S3.

### Protein extraction, immunoblotting and quantification

Zein and non‐zein proteins were extracted as previously described (Zhang *et al.*, 2019). Total seed proteins from wheat and maize were extracted with 12.5 mm sodium borate and 5% SDS buffer either containing 2% 2‐mercaptoethanol (reducing conditions) or without 2‐mercaptoethanol (non‐reducing conditions).

The immunoblotting procedure was described previously (Zhang *et al.*, 2019). The three primary antibodies used were mouse anti‐FLAG monoclonal antibody (Sigma‐Aldrich, F1804, St. Louis, MO, USA), rabbit anti‐GFP monoclonal antibody (Thermo Fisher Scientific, G10362) and chicken anti‐wheat gluten polyclonal antibody (Agresera, AS09571). The secondary anti‐GFP and anti‐FLAG antibodies were included in the kit of the ECL Western Blotting analysis system. The secondary anti‐wheat gluten antibody was purchased from Sigma‐Aldrich (Cat. 12‐341). The Etglo3 and zein proteins in Coomassie Brilliant Blue (CBB)‐stained SDS‐PAGE were quantified using AlphaView software (Alpha Innotech Corp., San Leandro, CA, USA).

### LC‐MS

Specific bands were excised from the CBB‐stained protein gel and sequenced by LC‐MS at the Biological Mass Spectrometry Facility of the Robert Wood Johnson Medical School of Rutgers University. The identified peptides were submitted to the Protein Knowledgebase (UniProtKB) to search for target proteins.

### TEM and immunogold labelling TEM

For TEM, 2‐ to 5‐mm‐thick sections of developing endosperm tissues at 20 DAP were cut with a Leica EM UC‐6 ultramicrotome and fixed overnight in 0.1 m sodium cacodylate buffer (pH 7.4) containing 2.5% glutaraldehyde. Fixed samples were processed in the Core Imaging Lab of the Robert Wood Johnson Medical School, Rutgers University and were imaged with a JEOL 1200EX electron microscope using an AMT‐XR41 digital camera.

For immunogold labelling TEM, thin slices of 20‐DAP endosperm tissues of *Etglo3* transgenic plants were fixed in the PBS buffer containing 4% paraformaldehyde. Samples were processed at the Bio‐Imaging Center of the Delaware Biotechnology Institute, including high‐pressure freezing/freeze substitution, sectioning and immunolabelling with the primary antibodies against GFP (ABCAM ab6556), FLAG (SIGMA F7425), 22‐kDa α‐zein (ABclonal A16746) and 27‐kDa γAzein (ABclonal A16745), followed by imaging.

### Yeast two‐hybrid assay

The yeast two‐hybrid assay was performed as described in the manual of the Matchmaker Gold Yeast Two‐Hybrid System (Clontech Cat. 630489). The binding domain (BD) plasmids pGBKT7‐Etglo3 and pGBKT7‐GFP were generated by cloning the signal peptide‐deletion open reading frame (ORF) regions of *Etglo3* and *GFP* in‐frame with the GAL4 BD. The activation domain (AD) vectors of pGADT7‐*zein*/*Etglo3*/*GFP* were constructed by inserting the signal peptide‐deletion ORF regions of *zein*, *Etglo3* or *GFP* to the 3ʹ end of the Gal4 AD. All primers are listed in Table S3.

### Bimolecular fluorescence complementation assay

The ORF of *Etglo3* was cloned into JW771 [containing the N‐terminal half of luciferase (NLUC)] to produce the construct Etglo3‐NLUC, whereas the ORFs of *Etglo3* and all *zein* genes were cloned into JW772 [containing the C‐terminal half of luciferase (CLUC)] to produce the constructs Etglo3‐CLUC, δ10‐CLUC, β15‐CLUC, γ16‐CLUC, α19‐CLUC, α22‐CLUC, γ27‐CLUC and γ50‐CLUC. The *Agrobacterium*‐mediated transformation of *Nicotiana benthamiana* leaves and luciferase detection were described previously (Zhang *et al.*, 2019). All primers are listed in Table S3.

## Author contribution

Z.Z., Y.W. and J.M. designed research; Z.Z., Y.D. and W.Z. performed research (Z.Z. did most experiments; Y.D. did TEM of wheat endosperm, performed BiFC and developed antibodies against zeins; W.Z did TEM experiment of teff endosperm); Z.Z., Y.W. and J.M. analysed data; and Z.Z., Y.W. and J.M. wrote the paper.

## Conflict of interest

The authors have no conflict of interest to declare.

## Supporting information


**Figure S1** Phylogenetic analysis of prolamins and α‐globulins from wheat, maize and teff.
**Figure S2** Alignments of α‐globulins from wheat, maize, rice, sorghum and teff.
**Figure S3** PCR verification and real‐time qPCR quantification of *Etglo3* in transgenic lines.
**Figure S4** Distribution of immunogold particles marking the FLAG tag within the electron‐dense (ED) aggregates and protein bodies (PBs) in endosperm cells of *Etglo3* transgenic plants.
**Figure S5** Immunoblotting using the anti‐FLAG antibody for polymerization analysis of Etglo3 and Etglo4 in mature transgenic maize seeds.
**Figure S6** Yeast two‐hybrid assay to test the interaction between Etglo3 and zeins, and between GFP and zeins.
**Figure S7** Real‐time qPCR quantification of gene expression related to the accumulation and trafficking of storage proteins in the developing endosperm of *Etglo3* transgenic plants.
**Table S1** The number of 20 types of amino acids in cereal α‐globulin proteins.
**Table S2** LC‐MS result of the top‐30 identified proteins on the top of SDS‐PAGE in Figure 5a.
**Table S3** All primers used in this study.
