# Supplementary material for: Towards coeliac‐safe bread
Source: Plant Biotechnol J. 2019 Dec 24;18(4):1056–65. doi: 10.1111/pbi.13273 (PMC7061869; doi:10.1111/pbi.13273)
Supplement: Supplementary file 1 — Figure S1 Phylogenetic analysis of prolamins and α‐globulins from wheat, maize and teff. Figure S2 Alignments of α‐globulins from wheat, maize, rice, sorghum and teff. Figure S3 PCR verification and real‐time qPCR quantification of Etglo3 in transgenic lines. Figure S4 Distribution of immunogold particles marking the FLAG tag within the electron‐dense (ED) aggregates and protein bodies (PBs) in endosperm cells of Etglo3 transgenic plants. Figure S5 Immunoblotting using the anti‐FLAG antibody for polymerization analysis of Etglo3 and Etglo4 in mature transgenic maize seeds. Figure S6 Yeast two‐hybrid assay to test the interaction between Etglo3 and zeins, and between GFP and zeins. Figure S7 Real‐time qPCR quantification of gene expression related to the accumulation and trafficking of storage proteins in the developing endosperm of Etglo3 transgenic plants. Table S1 The number of 20 types of amino acids in cereal α‐globulin proteins. Table S2 LC‐MS result of the top‐30 identified proteins on the top of SDS‐PAGE in Figure 5a. Table S3 All primers used in this study. [file PBI-18-1056-s001.docx]

**Supporting information**


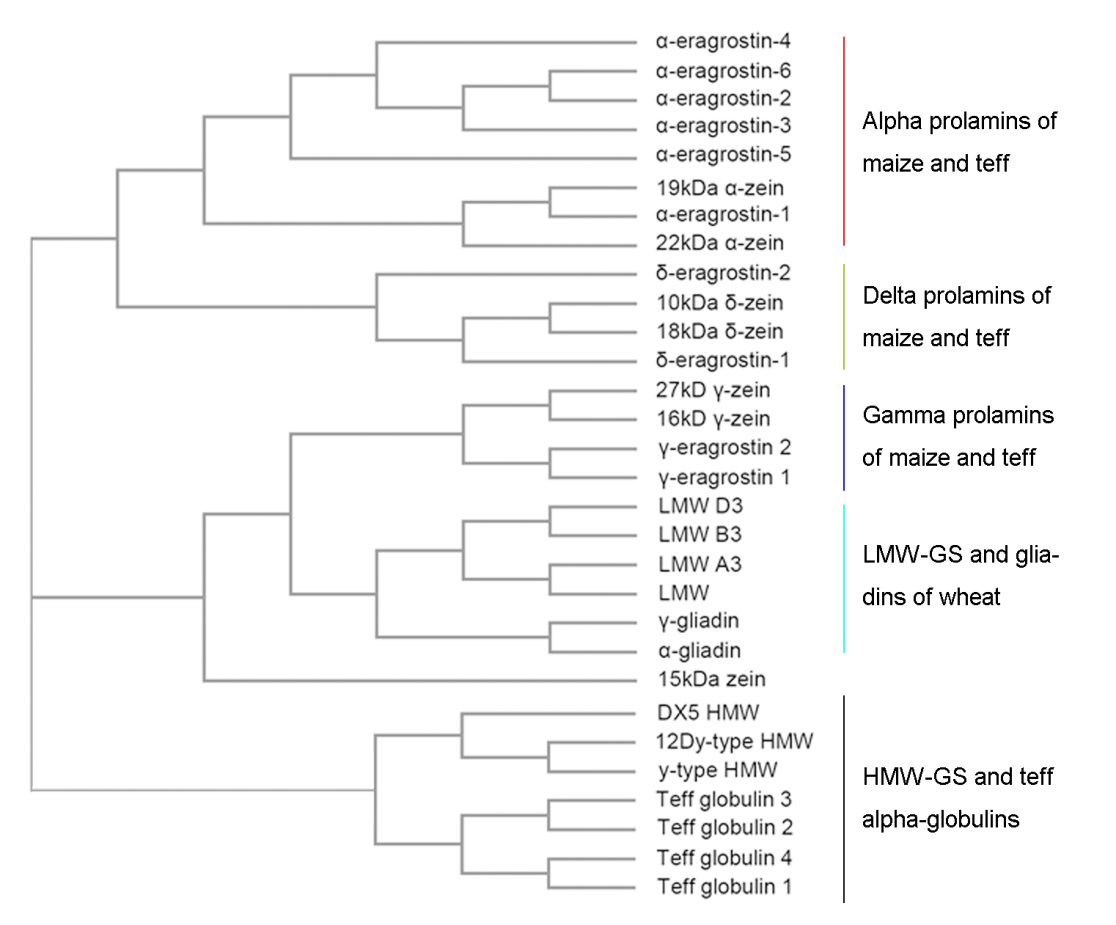


**Figure S1.** Phylogenetic analysis of prolamins and α-globulins from wheat, maize, and teff. Note: This is a neighbour-joining tree without distance corrections. The sequence information for these genes is taken from previous publications (Zhang *et al.* 2019; Xu and Messing 2009).


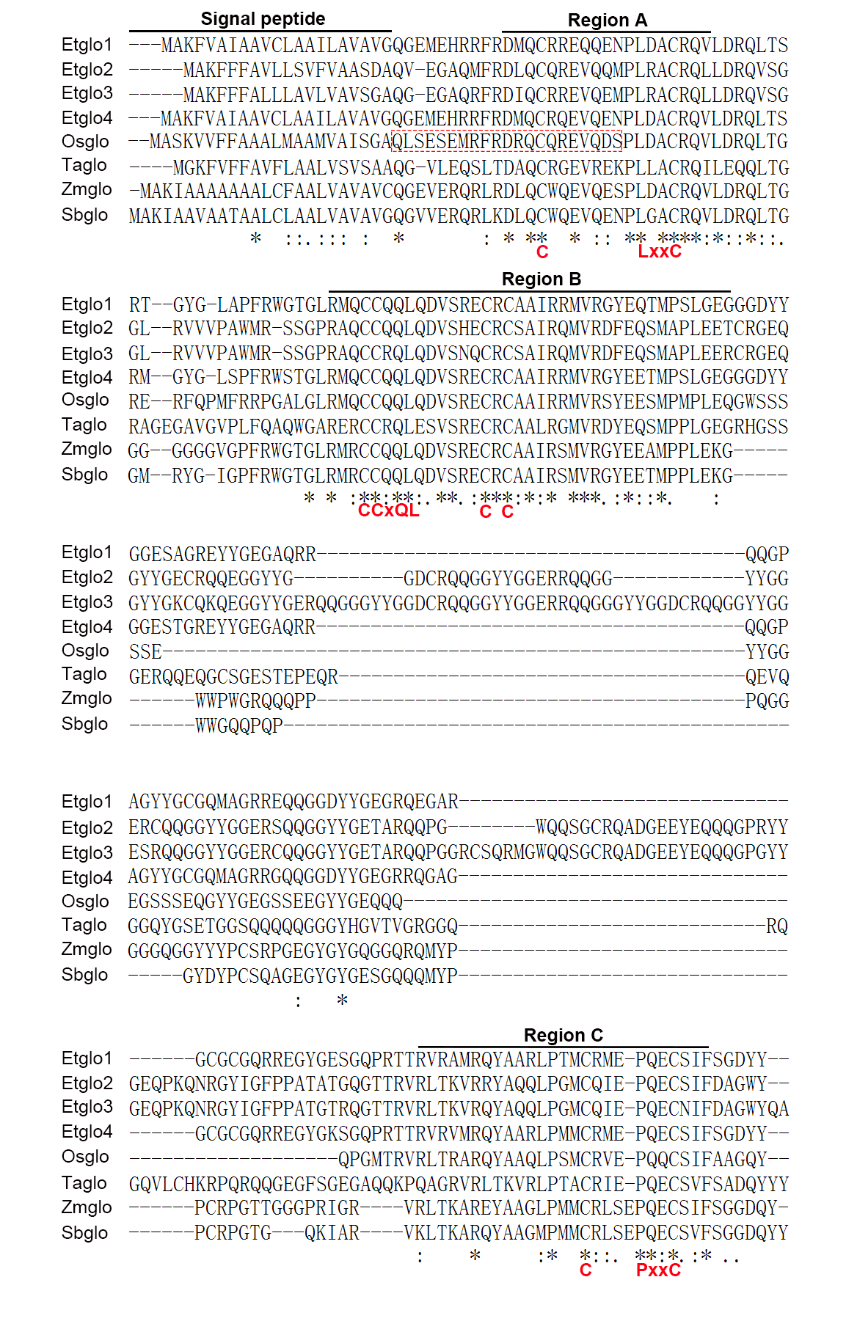


**Figure S2.** Alignments of α-globulins from wheat, maize, rice, sorghum, and teff. The ABC domains are highlighted as Regions A, B, and C. The eight conserved cysteine residues and three consensus motifs (LxxC, CCxQL, and PxxC) are marked by red letters.


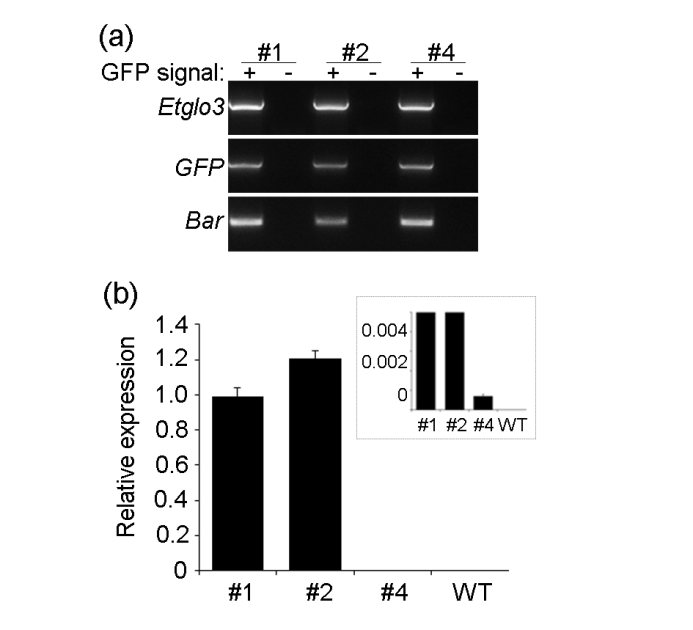


**Figure S3.** PCR verification and real-time qPCR quantification of *Etglo3* in transgenic lines. (a) Genomic DNA PCR validation of the three transgenic events. The signs “+” and “−” indicate the seeds per event with and without GFP fluorescence, respectively. (D) The real-time qPCR quantification of the expression level of *Etglo3* in the 20-DAP endosperm of the three transgenic lines. The data represent three biological replicates per event. The enlarged chart in the top left corner shows the level of *Etglo3* expression in Etglo3#4.


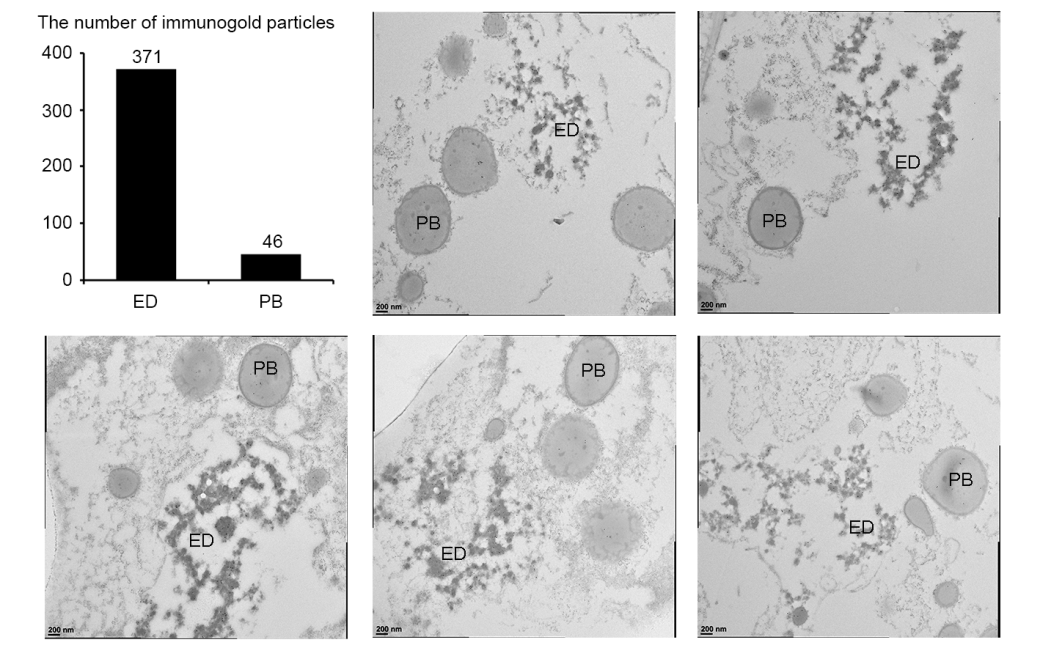


**Figure S4.** Distribution of immunogold particles marking the FLAG tag within the electron-dense (ED) aggregates and protein bodies (PBs) in endosperm cells of *Etglo3* transgenic plants. The immunogold particles were quantified in the five images. The numbers in the chart indicate the number of immunogold particles.


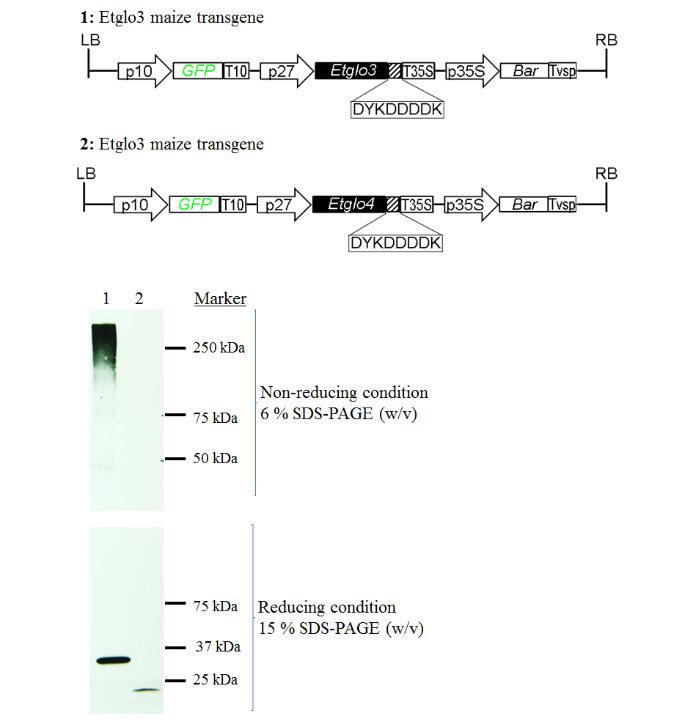


**Figure S5.** Immunoblotting using the anti-FLAG antibody for polymerization analysis of Etglo3 and Etglo3 in mature transgenic maize seeds. The upper panel schematically depicts the *Etglo3* transgene construct. Non-reducing condition means that extraction and loading buffer did not contain β-mercaptoethanol, whereas that for reducing conditions contained β-mercaptoethanol. The amount of total seed proteins loaded in each lane was 20 µg.


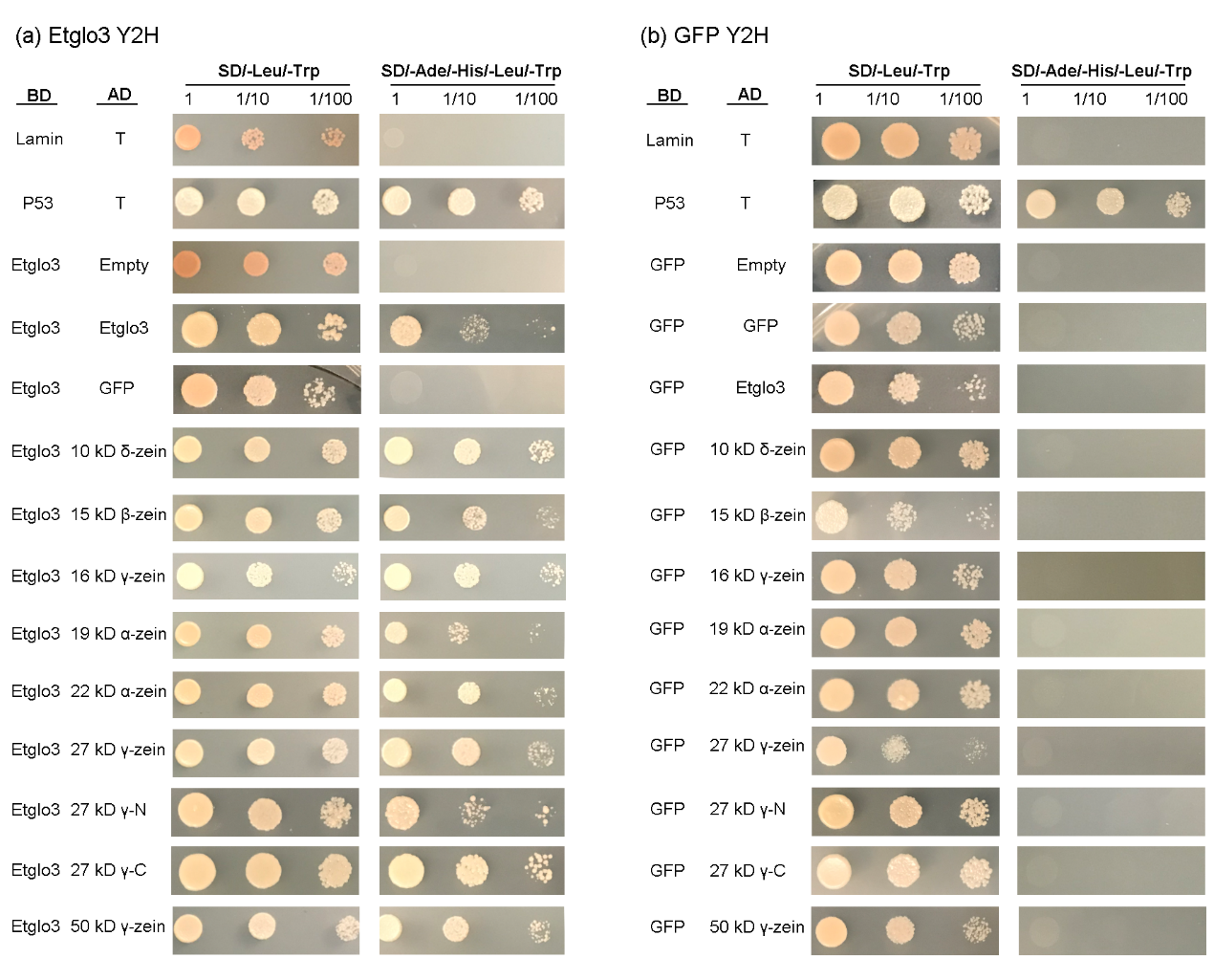


**Figure S6.** Yeast-two-hybrid assay to test the interaction between Etglo3 and zeins, and between GFP and zeins. BD, binding domain; AD, activating domain. The interaction between P53 and T-antigen (T) was used as a positive control, and the interaction between lamin and T-antigen was used as a negative control. The three gradient dilutions were 1, 1/10, and 1/100.


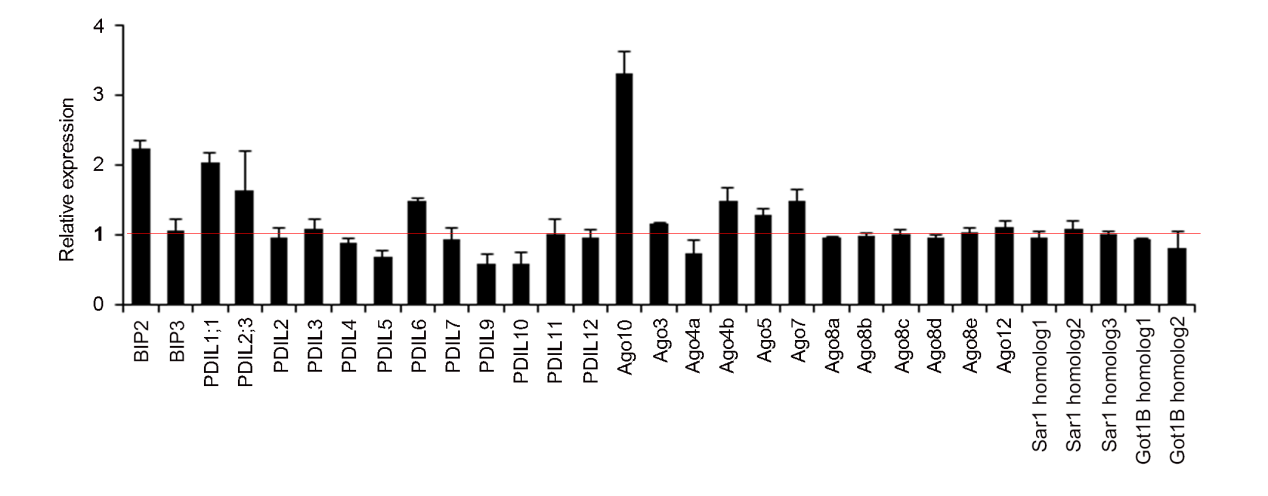


**Figure S7.** Real-time qPCR quantification of gene expression related to the accumulation and trafficking of storage proteins in the developing endosperm of *Etglo3* transgenic plants. The baseline “1” (red dotted line) represents the expression level of genes in the endosperm of Hi-A×B. The data shown are the means ± SD from three replicates of 20-DAP endosperm.

**Table S1.** The number of different amino acids in cereal α-globulin proteins.

Note: Etglo = teff α-globulin; Zmglo = maize α-globulin; Osglo = rice α-globulin; Taglo = wheat α-globulin.

**Table S2.** The top 30 identified proteins identified by LC-MS from the top of the SDS-PAGE gel in Figure 5A.

**Table S3.** List of primers used in this study.
